# Designing a Visit Schedule for Longitudinal Studies in Pediatric Research

**DOI:** 10.3389/fped.2022.883994

**Published:** 2022-09-12

**Authors:** Steven Hirschfeld, Michael Dellarco, Cindy J. Nowinski, Jerry Slotkin

**Affiliations:** ^1^Department of Pediatrics, Uniformed Services University of the Health Sciences, Bethesda, MD, United States; ^2^Whiting School of Engineering Johns Hopkins University, Baltimore, MD, United States; ^3^Department of Medical Social Sciences, Northwestern University Feinberg School of Medicine, Chicago, IL, United States; ^4^Center for Health Assessment Research and Translation, University of Delaware, Newark, DE, United States

**Keywords:** longitudinal study, birth cohort, measurement, pediatric, child health, child development, visit schedule, developmental trajectory

## Abstract

A challenge for longitudinal studies is combining individual assessments into visits that are scientifically logical, not burdensome for participants, well-choreographed, and operationally feasible. The visits then need to be sequenced and spaced to address the scientific goals and generate a data archive that is sufficiently robust and well-documented to support subsequent analyses. This paper summarizes comprehensive multi-disciplinary activities that were coordinated to design the content, format, and structure of the National Children's Study and concurrently serve as a model and resource for other studies.

## Introduction

The National Children's Study (NCS) was proposed as part of the Children's Health Act of 2000, which authorized a national longitudinal study of environmental influences on children's health and development that included three directives.

1. Incorporate behavioral, emotional, educational, and contextual consequences to enable a complete assessment of the physical, chemical, biological and psychosocial environmental influences on children's wellbeing.
2. Gather data on environmental influences and outcomes on a diverse population for children, which may include the consideration of prenatal exposures.
3. Consider health disparities among children, which may include the consideration of prenatal exposures.

What follows is a discussion of how the NCS considered the broad mandate incorporated into the Children's Health Act and integrated these considerations in developing assessments, combining assessments into individual visit content, and designing a visit schedule that would be comprehensive and flexible but not burdensome.

The general model for longitudinal studies is to proceed in waves or cycles with alternating periods of activity, analysis, design, and implementation. The NCS was unique in two regards- ongoing without interruption and using a pilot study to test and refine measures, then moving the measures into the larger study to attain continuous data collection. Our intent is to describe and highlight this new model.

The NCS evolved a plan that would identify the factors and influences that lead to a healthy, functional 21-year-old from before birth and, concurrently, identify factors and influences for a series of high-impact public health conditions that positively and negatively affect children, their families, and their communities.

The need for ongoing development and testing of age-appropriate and informative assessments was the primary rationale for establishing an ongoing pilot study that would run 2–3 years in advance of any particular stage of the larger main study. New methodologies could be properly validated in the populations of interest, in contrast to pulling measures “off the shelf.” Using measures from other studies or even clinical practice might forego the opportunity to gain the precision and specificity needed to inform the trajectories and outcomes and perform the integrated analyses the NCS planned.

A sampling of high-impact public health conditions was selected based on prevalence and estimated lifetime health costs, including those affecting immediate household members, other family, and communities.

These conditions are summarized in Table 1.

**Table 1:** Prevalence estimates per 100,000 for selected childhood illnesses or medical conditions^*^.

Obesity—ages 2–19 (1)	17,000
Premature birth (2)	12,200
Attention deficit/hyperactivity disorder—ages 3–17 (3)	9,500
Asthma–reactive airway diseases—ages 0–18 (4)	9,300
Learning disorders (aggregate)—ages 3–17 (5)	8,000
Birth defects (aggregate) (6)	3,000
Autism spectrum disorders (aggregate)—ages 6–17 (7)	2,000
Schizophrenia variants—cumulative (8)	1,100
Congenital heart disease (aggregate) (9)	1,000
Epilepsy (aggregate)—ages 0–17 (10)	600
Childhood cancers (aggregate)—ages 0–19 (11)	250
Trisomy 21 syndrome (12)	120
Fragile X syndrome (13)	25

*Note that the legal federal threshold for a rare disease currently is a prevalence of about 64 per 100,000.

*Table Reverences*

*1. http://www.cdc.gov/obesity/data/childhood.html*

*2. Hamilton BE, Martin JA, Ventura SJ. Births: Preliminary data for 2009. National vital statistics reports web release; vol 59 no 3. Hyattsville, MD: National Center for Health Statistics 2010.*

*3. http://www.cdc.gov/nchs/data/series/sr_10/sr10_258.pdf*

*4. http://www.cdc.gov/nchs/data/series/sr_10/sr10_258.pdf*

*5. http://www.cdc.gov/nchs/data/series/sr_10/sr10_258.pdf*

*6. http://www.cdc.gov/ncbddd/birthdefects/data.html*

*7. http://www.cdc.gov/ncbddd/autism/data.html*

*8. http://www.cdc.gov/mentalhealth/basics/burden.html*

*9. http://www.cdc.gov/ncbddd/heartdefects/data.html#References*

*10. http://www.cdc.gov/epilepsy/basics/fast_facts.htm*

*11. http://surveillance.cancer.gov/prevalence/canques.html*

*12. http://www.aap.org/en-us/about-the-aap/aap-press-room/pages/Down-Syndrome-Prevalence-in-the-~United-States.aspx*

*13. http://www.fragilex.org/fragile-x-associated-disorders/prevalence/*

The prevalence of many of the conditions in Table 1 is possibly underestimated due to disparities in health and access to health care, therefore limiting the opportunity for diagnosis. Children with less severe symptoms or with restricted access to health care may have impacts on their health from these conditions but these impacts do not rise to a level of magnitude captured by epidemiological surveys or formal health care records.

For uncommon and rare conditions such as childhood leukemia or Level 1 Autism Spectrum Disorder, a study of initial cohort size of 100,000 participants is either on the border or unlikely to have sufficient power to make definitive exposure-outcome associations as a standalone study. To increase power for uncommon and rare conditions and provide additional context for other observations, the NCS formed an alliance with other ongoing or planned large longitudinal birth cohort studies internationally to pool data and align collection methods and variable names (1).

Using an initial enrollment target of 100,000 children and assuming an annual retention rate of 97 percent, which the NCS was able to meet over a 5-year period, at the end of 21 years a study would have a sample size of approximately 54,000 participants (2).

### Health Phenotype Concept

Each participant was planned to be assessed at each encounter using a health phenotype framework with the following rationale:

1. Use a conceptual framework grounded in health measurement that always applies to all study participants.
2. Capture a broad scope of outcomes that cover the complexity of human health and do not limit observations to presence or absence of any particular conditions or diseases.
3. In addition to noting associations between negative or limiting exposures and outcomes, identify enabling exposures and conditions that can improve functioning, adaptability, and thriving.
4. Establish consistency in reporting outcomes and exposures. Research fields have different methods, paradigms, and levels of precision. An overall framework can help relate the different types of assessments and data collection methods to one another.
5. Maintain flexibility as new opportunities and assessment innovations arise so they can be integrated into the conceptual framework.
6. Maintain sustainability, so as analytical contexts and methods evolve and new concepts emerge, resources are available to include them in the conceptual framework.

Historically, the concept of health evolved to include multiple dimensions. Over the last century, population-level health statistics have demonstrated marked progress in childhood survival with decreases in infant mortality, including a decrease in many other measures of morbidity and mortality. Morbidity and mortality are important indicators to monitor, but are not comprehensive assessments of the complex dynamics that constitute children's health. For a more detailed discussion, please see the papers on the PRISM model (3, 4).

The Institute of Medicine report on the NCS from 2008 noted a dearth of instruments to assess the various dimensions of health (5). The work of the Health Measurement Network specifically addressed this point in the development of instruments and the subsequent construction of visit structure and schedule.

### Linking Health Phenotype to Specific Diseases and Conditions

The NCS was designed as a study and therefore was not intended to be a national platform for medical screening, nor a vehicle to provide direct care or substitute for the health care delivery system. As such, the goal was to directly collect information regarding observations and events on all participants, but not focus on classifying participants into predetermined disease categories. Many medical diagnoses are not stable variables and the classification systems for diseases and conditions are dynamic and regularly revised. Providing a focus on primary observations enables analysts and future researchers to apply their own criteria for phenotypic and diagnostic classification.

Using reactive airway disease as an example, the NCS emphasized accurately capturing medical history and events, participant experiences, and respiratory symptoms, coupled with biospecimens, genetic analyses, records of any interventions and environmental samples. Researchers can then use these data with the case definitions and classifications they consider relevant for their analyses.

Specifically, someone may want to examine exposures that trigger symptoms, such as wheezing after exercise or wheezing after upper respiratory infections coupled with coughing at night. The analyst may furthermore want to match that with people who repeatedly consult with health care providers for shortness of breath and possible treatment. The NCS would collect and capture all these factors. Analysts could select one outcome, or any combination of outcomes, without requiring a formal diagnosis to make the exposure-outcome associations.

This approach is consistent with the rapidly evolving conceptualization of disease taxonomy as described by the National Research Council in the 2011 monograph, “Toward Precision Medicine” (6).

The need for flexible data collection systems based on primary observations and assessments can support rapid updating of classification or nosology in response to advances in biological and clinical knowledge.

### Considerations Regarding Signal Generation and Capture

Even in an intensive care unit with continuous monitoring, gaps in critical information limit what can be gathered in a timely manner. Some of the factors include:

- the sensitivity, specificity, and receiver operator characteristics of instruments
- the modalities of measurement such as pressure, chemoconcentration including pH, electrical activity, positional change and movement
- the analytic capability to distinguish signal from noise
- inability to detect or capture a function of interest
- legal, technical, and ethical limits on what degree of invasiveness, whether it is physical, psychological, or involves other privacy factors, is feasible and permissible

The general characteristics of signal processing from an engineering perspective are to mathematically describe the sensitivity, or ability to detect a signal of interest, the specificity, or ability to discriminate a signal of interest from other signals or noise, and the receiver operator characteristics, or signal range and thresholds for the signal detection system.

The natural world does not generally consist of pure signals, but rather consists of composite and complex signals that require detection and processing to interpret. These signals are subject to bias, uncertainty and, consequently, imprecision.

Signal detection and outcome assessment are performed using intermediaries such as physical instruments, questionnaires, images, or other observations, all of which function as filters and introduce bias and imprecision. All intermediaries introduce a cost that should be factored into interpreting outcomes. Whether that cost is quantitatively determined or tacitly acknowledged or ignored, the blunting and blending of assessments affects the precision and bias of measurement.

Health outcomes research, including the detection, assessment, and progress of disease, generally does not have pathognomonic assessments that can be quantified with high precision. One exception is all-cause survival.

The concept of overall health does not have a universally accepted definition that readily lends itself to measurement. Partitioning phenotype and health into domains and subdomains is one approach for making assessments and linking them to outcomes, with the implication that what is measured is in some way related to pathologic progression of a disease or medical condition or benefit or harm from an intervention or exposure. There is a skew in clinical research toward either defining a statistical norm or assessing processes that have a negative health impact, such as diseases or known medical conditions. There are few reliable general indicators of thriving. The NCS attempted to address this challenge by considering the use, or if need be, the development, of assessment tools that could capture information across a broad range of phenotypes and health conditions at all stages of development.

## Methods

In 2011, the NCS founded a Health Measurement Network with two major goals:

- Develop a concept of health that could be measured across the relevant age and developmental spectrum.
- identify methods and instruments to assess health that are age- and developmental stage-appropriate.

The NCS Health Measurement Network reached consensus that:

- Health is a multidimensional concept.
- Each dimension can be assessed along a continuum from very low to very high levels.
- Each dimension includes multiple domains.
- Each domain can be assessed *via* multiple measurement modalities.
- Health develops over time and in response to a complex and dynamic set of child-environment interactions.

In parallel with the methods analysis and research, the group regularly discussed the operational challenge of assembling a series of measurements to choreograph each individual study visit so that it would be informative but not burdensome, and then a schedule of study visits to capture critical information at opportune times during child development.

The NCS proposed to use two concepts, phenotype and profile, to describe each participant. The term *phenotype* is used for the observable characteristics including morphology, physiology, developmental stage, behavior, and products of behavior. Phenotype results from the interaction of genetics and environment.

The NCS developed a framework for data collection based on the legal and scientific mandate to analyze health and development. In the proposed data collection framework, one axis is health and another axis is human development; thus, any phenotypic description will have both a health and a development component. The framework guides the development of assessments and the structure of data collection to ensure that essential and relevant information to understand health and development are included.

The term *profile* is used for the larger concept of phenotype as the product of genetic and environmental influences, while specifically describing and assessing environmental context. A *profile* includes observable characteristics about the participant as phenotype plus the contextual information about the environment, such as air particle measures, noise level, family structure and dynamics, access to health care, etc. A phenotype is a subset of a profile that describes an individual, while a profile includes the characteristics of the individual plus the environmental context.

The NCS perspective was that health is a positive concept and each child has a unique profile of health strengths and deficits. Impairment in any domain is not equated with ill health. For example, a child with a physical mobility limitation should not be considered unhealthy, because she may have a wide array of health strengths in other domains and may have the resources to adapt to mobility challenges.

Within this paradigm, child health is characterized by a multi-dimensional profile rather than a single number or category such as poor, good, or excellent. These profiles are assessed serially over the course of the study to describe trajectories of health. The proposed approach acknowledged the complexity of health.

### Content Development

Outcome measure or endpoint properties ideally:

- Are quantitative over an informative range
- Change direction with underlying condition status
- Change proportionately and predictably

A caveat is that a readout with mathematical precision may not have biological significance or even precision for the underlying measure.

Each candidate measure was evaluated based on these criteria, with limits and compromises noted.

The entire content development process was intended by design to be compatible with a machine-readable environment to lower costs, improve consistency and reliability, and produce documents and instruments faster than other alternatives.

The core questionnaire and all the modules had a similar design process. Specific domains were proposed by individuals or working groups, vetted through subject matter experts, and then selected for possible inclusion in a study visit.

Once a domain was selected, proposed items to assess components of the domain were drawn from several sources, including a library of items and modules from other studies, previous field experience, and domain experts from the Health Measurement Network.

Candidate items were then assembled into draft instruments. Each instrument was adjusted through a visit choreography team to determine sequence and flow, including biospecimen and environmental sample collection, observation time, and other factors and activities. The draft visit was then submitted for regulatory and scientific review.

Following regulatory clearance, all aspects of the data collection process were field tested in the pilot phase and subsequently evaluated for feasibility, acceptability, and cost. The field testing, in turn, informed the next cycle of content development.

One example of the use of pre-existing instruments was the integration, adaptation, and reciprocal development of material conducted within the collaboration between the NCS and the NIH Toolbox® (NIHTB) initiative. The NCS extended the lower limit of the age range of several NIHTB assessments and adapted NIHTB instruments to be available on portable devices such as iPads for field use. The NCS was able to fund new instruments and modifications of other NIHTB instruments to address the ages and goals of planned study visits.

The NCS also collaborated with the European Commission-funded methods development initiative, called Global Research in Pediatrics, to develop harmonized terminology for pediatric concepts and terms, data collection and analytic techniques. Reports on these efforts were submitted to and accepted by the European Commission and posted on the internet (7).

**Figure 1:**
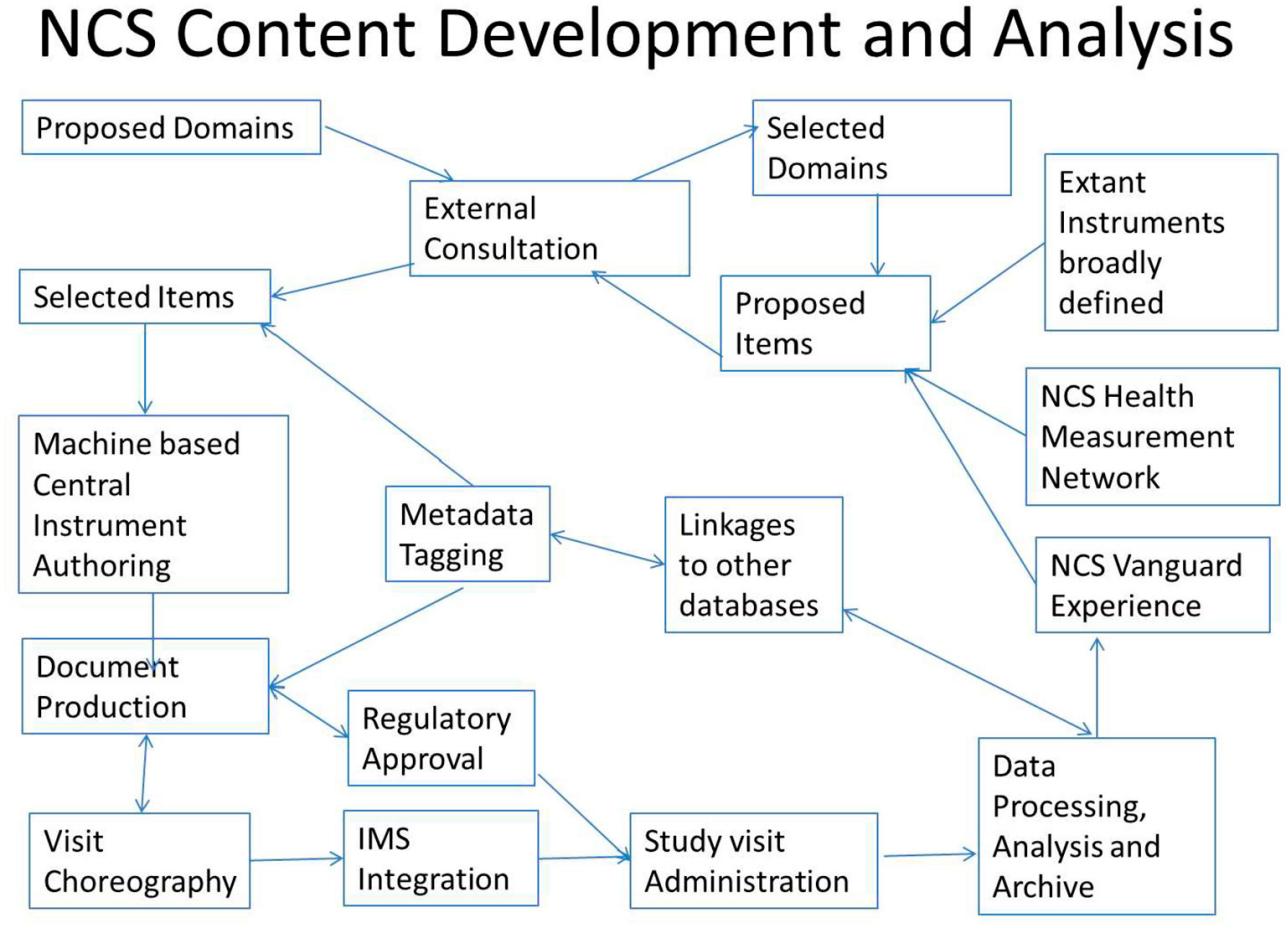
Study content development cycle. IMS, Information Management System.

The process is summarized in the following schematic Figure 1, with the cycle beginning in the upper left corner with Proposed Domains.

A study operations team assigned the primary collected data fields additional metadata tags containing additional information to enable sorting, archiving, and linking of the collected data to other data sources for analysis. The metadata tags are flexible, so that as new classifications or nosology evolves, the collected study data can be retagged and remapped to remain informative without altering the primary data.

Through the metadata tagging, all health phenotype data can readily be mapped to common classifications, such as any version of the International Classification of Diseases (ICD), Medical Dictionary for Regulatory Activities (MedDRA) or the Systemized Nomenclature of Medicine (SNOMED).

In addition, the NCS commissioned the development of a comprehensive cross-linking tool to interrogate over 100 federal databases from domains such as economics, natural resources, education, health, crime and justice, demographics, climate, housing, environmental sampling, etc. These additional data can be linked primarily based on geographic coordinates or location and time and, when feasible and appropriate, based on population and economic demographics, to provide further context to already planned direct data collection from participants and their environment and the associated administrative data collection, such as school and health records.

## Results

### Proposed Study Visit Schedule

The NCS emphasized data collection early in pregnancy when feasible and early in child development in all cases. Consequently, pregnancy data collections in the proposed plan were scheduled, if possible, prior to approximately 20 weeks gestation and once again later in pregnancy. Data collections for children would be scheduled at birth and 3 additional times during the first year, then 9 times until 5 years old, for a total of 13 opportunities for data collection from birth until 5 years. Seven of the opportunities would be face-to-face encounters and include biospecimen and environmental sample collection. The other six would be remote data collections, typically by telephone or virtual meeting interview and, if feasible, supplemented with remote data entry, including an option for wearable devices. Scheduling most data collections within the first 5 years would both address critical knowledge gaps and maximize data collection while retention of participants is highest.

Subsequent data collections would occur about every other year until 21 years of age, for a total of 8 additional data collection opportunities. In sum, about 21 data collection opportunities per child were planned, with changes possible based upon experience, scientific opportunity, logistical factors, and resources available.

The proposed visit schedule is deliberately flexible, in that children would not have assessments administered precisely at a given age, but instead, within a window of several weeks during the early years to months around the later years to improve compliance and to capture data across a range of ages. An analogous concept is population pharmacokinetic analysis sampling of different individuals at different times.

Table 2 summarizes the schedule.

**Table 2:** Proposed visit schedule.

**Prenatal**	**Infant**	**Pre-school**	**Youth**
**Pregnancy before 20 weeks**	**Birth**	*18 months*	**6–7 years**
**Pregnancy after 20 weeks**	*4 months*	**21 months**	**8–9 years**
	**8 months**	*30 months*	**10–11 years**
	*11 months*	**36 months**	**12–13 years**
	**14 months**	*42 months*	**14–15 years**
		**48 months**	**16–17 years**
		*54 months*	**18–19 years**
		**60 months**	**20–21 years**

*Visits in bold are in person and visits in italics are entirely remote.*

*The Youth visits can occur any time in the 2 year period as an in person visit with a remote option as assessment technology advances.*

*Total number of data collection is up to 23 of which 6 are entirely remote and others may become remote. Of the 23 visits, 2 are prenatal, 5 are during infancy, 8 are during preschool ages, and 8 are as youth. The weighting is toward the earlier years where the largest data gaps exist.*

### Study Visit Format

To manage participant burden, individual health assessments must be rapid, accurate, and affordable. To reach these goals, the visit plan consisted of:

- A core questionnaire given to all participants at each visit to assess the general health phenotype.
- Supplemental questionnaires with modules on individual topics. The topic-specific modules are based on contextual triggers such as age at time of administration, a new diagnosis, arrival or departure of a household member, or a particular exposure. In addition, some participants who did not trigger a supplemental module were to be selected at random to respond to the same questions, to ensure the validity of the supplemental questions, provide some internal controls, and recognize that some triggers may be missed.

Using modules increases flexibility and can reduce the overall data collection burden.

In addition to questionnaires, biological specimens, and environmental samples, other modalities for data capture such as sounds, images, wearable monitors that provide motion and physiologic readouts, geographic movements, and other dynamic data were planned. In addition, mapping of social interactions and networks through a combination of questionnaires and geographic movements were planned to address topics related to decision-making, degrees of social engagement, influences and influencing, and isolation. The core and supplemental questionnaires combined with the domain-specific assessments were essential to calibrate the data from other modalities and to link collected study data to other data sources.

Table 3 summarizes the planned domains and timing up to age 21 years. The NCS Vanguard Study tested visits up to age 4 before the program ended, so no data are available for the later planned visit structures. The experience up to the age 4 visit was general acceptance by participant families of the visit content.

**Table 3:** Composite visit schedule and assessments with estimated timing (in minutes).

**Domain**	**Visit type**	**Birth**	**4 M**	**8 M**	**11 M**	**14 M**	**18 M**	**21 M**	**30 M**	**36 M**	**42 M**	**48 M**	**54 M**	**60 M**	**6-7 Yr**.	**8-9 Yr**.	**10-11 Yr**.	**12-13 Yr**.	**14-15 Yr**.	**16-17 Yr**.	**18-19 Yr**.	**20-21 Yr**.
Cognition	Child in-person			15		15		15		30		26		35	20	30	20	25	20	20	20	22
*Cognition/future option*	*Child remote*																					
Cognition	Parent in-person					35																
Cognition	Parent remote				15	15																
Motor	Child in-person			5		5		5		14		20		17	22	20	18		10		8	10
*Motor/future option*	Child remote																					
Motor	Parent in-person							15														
Motor	Parent remote		5		5		5															
Sensory	Child in-person			8		3		8		14		10		14	18	17	15	13	15	13	15	13
*Sensory/future option*	*Child remote*																					
Sensory	Parent in-person																					
Sensory	Parent remote				3				5		2		5									
Social/emotional/behavioral	Child in-person			18		18		6		12		0			20							
Social/emotional/behavioral	Child remote															19	16	20		30	24	8
Social/emotional/behavioral	Parent in-person			18		18		6		12		0										
Social/emotional/behavioral	Parent remote		21	14	13	10	15	4	13	12	16	0	19	3	22	17	14	2	9	12	6	6
Environmental	Child in-person													15	15	15		15	15	15	15	15
Environmental	Child remote															10	13	25	25	24	38	22
Environmental	Parent remote		20	12	22	5	22	10		42		11	15	18	25	37	15	8	29	29	6	10
Environmental	Home – remote		7		7	121	7	99	7	114		99		114	84	114		114	114	114	99	114
Environmental	External				15					15				15		15			15	15		15
Physical health & systems review-child	Child in-person	15		11		31		14		19		32		22	27	40	23	38	45	22	42	37
Physical health & systems review-child	Child remote																		19	19	19	19
Physical health & systems review-child	Parent remote			5		12		18		20		20		19	17	15	19	17				
Physical health & systems review-parent	Parent in-person	15				15						15				15			15			
Physical health & systems review-parent	Parent remote	3		18		16		12		15		11		15	14	15	15	15	15	15	15	15
CORE Questionnaire	Parent remote	30	10	8	8	8	10	10	15	8	15	8	15	8	8	8	10	10	8	10	10	10
																						
Subtotal	Child in-person	**15**	**0**	**57**	**0**	**72**	**0**	**48**	**0**	**89**	**0**	**88**	**0**	**103**	**122**	**122**	**76**	**91**	**105**	**70**	**100**	**97**
Subtotal	Child remote	**0**	**0**	**0**	**0**	**0**	**0**	**0**	**0**	**0**	**0**	**0**	**0**	**0**	**0**	**29**	**29**	**45**	**44**	**73**	**81**	**49**
Subtotal	Parent in-person	**15**	**0**	**18**	**0**	**68**	**0**	**21**	**0**	**12**	**0**	**15**	**0**	**0**	**0**	**15**	**0**	**0**	**15**	**0**	**0**	**0**
Subtotal	Parent remote	**33**	**56**	**57**	**66**	**66**	**52**	**54**	**33**	**97**	**33**	**50**	**54**	**63**	**86**	**92**	**73**	**52**	**61**	**66**	**37**	**41**
Subtotal	Home – remote	**0**	**7**	**0**	**7**	**121**	**7**	**99**	**7**	**114**	**0**	**99**	**0**	**114**	**84**	**114**	**0**	**114**	**114**	**114**	**99**	**114**
Subtotal	External	**0**	**0**	**0**	**15**	**0**	**0**	**0**	**0**	**15**	**0**	**0**	**0**	**15**	**0**	**15**	**0**	**0**	**15**	**15**	**0**	**15**
	Cleanup					**40**						**40**				**40**			**40**			**40**
Total in-person+in home +external+cleanup (centrifuge, prep, shipping, etc.)		**30**	**0**	**75**	**0**	**180**	**0**	**69**	**0**	**101**	**0**	**143**	**0**	**103**	**122**	**177**	**76**	**91**	**160**	**70**	**100**	**137**
Environmental domain notes:	>Timing of recommended measures which are NOT included here: -Measures assumed to be in Core (e.g., Demographics, Diet, Housing&Neighborhood characteristics Qxs) -Biospecimen collection, medical condition, e.g., allergic status (assumed to be under Physical Health Domain) -Measures which overlap with Social/Emotional/Behavioral Domain (e.g., social determinants, sun exposure, physical activity) -Measures entirely external to participants (e.g., neighborhood obs, GIS/extant data analyses) >Where timing information is unknown for a given scale, 30 sec/item has been used, thus timings are considered high for child-remote and parent-remote. >The Enviro Domain Team has not yet fully considered data collection by lifestage (i.e., groups of ages in this table); thus measures currently recommended annually may be recommended less frequently in next version. >The Enviro Domain Team has not yet fully considered in-person versus remote data collection. Timings here assume any Qx that could be administered remotely is remote, and that sample collection is done by DCs (not self-collected by participants). >In-Home/External visit types include observations and/or sample collection by DC at time of visit.																					

*Visits marked in blue are entirely remote. As remote technology evolves, additional assessments can be added with slots reserved in grayed out rows. Comprehensive home visits were capped at 3 h. Environmental assessments include home, neighborhood, school, recreation, town or city, and regional. Physical Health & Systems Review included dietary intake, physical activity, illness, healthcare system encounters, medication use and other parameters noted in the cited papers on Environmental and Physical Health.*

## Discussion

The generation of a feasible visit schedule for a comprehensive longitudinal study is complex. A conventional approach is to perform the planning and implementation in waves as interim data and resources become available. The NCS was planned to be of such a scope both scientifically and logistically that activities would be ongoing continuously and the time needed to develop, test, calibrate, and evaluate new measures would not be feasible on an interim basis. Thus, the structure evolved to include two parallel, but time-frame-shifted activities, one a pilot, referred to as the Vanguard Study, to perform all the operational testing and development, and the other the main study to implement the processes and products of the pilot study scaled up to capture sufficient data about less common and even rare events with quality and precision.

The NCS experience established the feasibility of running a pilot study to advance scientific and logistical understanding prior to expending the resources for a large national study with a target enrollment of 100,000 participants. This was accomplished with an active enrollment of approximately 5,000 participants. As the planned Main study was never launched the pilot was terminated. Consequently a final array of visit schedules and content was not completed. What was completed were:

- Formation of a comprehensive longitudinal framework for a broad scope of child health and development that was not just limited to origins of diseases
- A dynamic process for specialty content development that was fit for purpose and did not rely on recycling existing instruments of varying quality and relevance
- A continuous data collection model using a pilot study with integrated proof of concept analysis
- A systematic comparison of recruitment processes that evaluated resource, efficiency, and bias profiles
- Systematic gap analyses of assessment tools and capabilities with adaptation or innovation or design of new instruments and modalities
- Use of data structures and formats that allow interoperability with other study data, adminstrative data, and data collected from the health care delivery system (real world data) that could effectively leverage the investment

Child development is multi-dimensional, and recognition and integration of approaches are required to produce datasets that are of value to current and future generations. The scope and challenge of the project required the establishment of a dedicated expert working group to identify gaps, propose new measures, and supervise the ongoing development. While the operational specifics of what and how could be measured years in advance is not feasible given the rapid evolution of technology, a framework for topics of interest and a proposed schedule using whatever could be anticipated using current technology could be used for planning. The advantage of the pilot study was that as technology, biological understanding, and research methodology including informatics and interoperability evolved, the platform could be continuously refreshed, updated, and properly calibrated.

The limitations of the NCS experience are primarily related to the limited duration of <5 years and the need to initiate data collection while concurrently developing the theoretical framework and the domain-specific visit assessments. Consequently, the empirical data to validate the proposed content and schedule were never generated. Instead, we have fragmented experience with selected measures and visit content and schedules that align with the proposed schedule. In addition, the precise mapping from the repertoire of proposed and available assessments remains a work in progress.

One possible limitation of the approach of using event triggers to initiate activation of a topic specific module is the accurate recognition of triggers to administer the topic-specific modules. Use of the wrong module or not having an appropriate module can result in lost opportunity and lack of precision. Thus, the random selection of additional study participants wihtout a trigger for a given module would help improve the process and interpretation of the data collection.

Another possible limitation is the content and structure of the core questionnaire to capture relevant information. The precision and comprehensiveness of the core questionnaire and modules are essential to provide high-level and accurate assessments of the phenotype of each child.

The scope of the NCS domains and the need to integrate methods across domains within the NCS and with other studies drove the establishment of the content development framework and process. The Health Measurement Network and the ongoing platform of the NCS to pilot and validate candidate assessments provided a unique and rigorous approach to harmonizing and improving the reliability and use of tools to measure outcomes for children. Part of the NCS vision was to provide a dynamic library of validated outcome measures for studies everywhere that planned to enroll children, and to become an international resource for advice and collaboration on method and assessment development.

The modular approach around a core questionnaire not only proved logistically feasible, but provides flexibility for analysis. The layered approach proposed in the study framework manuscript allows for interpretation of different assessments and tools at different ages to construct trajectories by mapping outcomes into different layers of complex domains. Specifically, assessment tools that shift with different ages and developmental stages can still be mapped to the same domain and the data can be interpreted in the context of other assessments. For example, an assessment that has purely observational data in infancy can be linked with data from an assessment that uses verbal responses at later stages using the layered domain approach. An analytic approach using normalized data expressed as a proportion along the receiver operator characteristics of a given assessment can be used to construct trajectories and build complex, higher-order models.

The principles and practices described above were designed to be modular and interoperable and thus may be applied as relevant to ongoing or future longitudinal studies.

## Data Availability Statement

The original contributions presented in the study are included in the article/supplementary material, further inquiries can be directed to the corresponding author/s.

## Author Contributions

All authors listed have made a substantial, direct, and intellectual contribution to the work and approved it for publication.

## Funding

Life Course Health Sciences (LHS) Working Group was supported by National Children's Study contracts to NORC at the University of Chicago (Contract Number: HHSN275201200004I; Task Order HHSN27500003) and Westat (Contract Number: HHSN275201200005I; Task Order HHSN27500003).

## Conflict of Interest

The authors declare that the research was conducted in the absence of any commercial or financial relationships that could be construed as a potential conflict of interest.

## Publisher's Note

All claims expressed in this article are solely those of the authors and do not necessarily represent those of their affiliated organizations, or those of the publisher, the editors and the reviewers. Any product that may be evaluated in this article, or claim that may be made by its manufacturer, is not guaranteed or endorsed by the publisher.

## References

[1] Etzel R Charles M-A Dellarco M Gajeski K Jöckel K-H Hirschfeld S. Harmonizing biomarker measurements in longitudinal studies of children's health and the environment. Biomonitoring. (2014) 1:6. 10.2478/bimo-2014-0006

[2] Guttmacher AE Hirschfeld S Collins FS. The National Children's Study–a proposed plan. N Engl J Med. (2013) 369:1873–5. 10.1056/NEJMp1311150 24224620 PMC5101954

[3] Hirschfeld S. Introduction and goals for the national children's study. Front Pediatr. (2017) 5:240. 10.3389/fped.2017.00240 29520353 PMC5827357

[4] Hirschfeld S Goodman E Barkin S Faustman E Halfon N Riley AW. Health measurement model-bringing a life course perspective to health measurement: the PRISM Model. Front Pediatr. (2021) 9:605932. 10.3389/fped.2021.605932 34178878 PMC8222802

[5] National Research C Institute Institute of Medicine Panel to Review the National Children's Study Research P. The National Academies Collection: Reports funded by National Institutes of Health. The National Children's Study Research Plan: A Review. Washington (DC): National Academies Press (US)

[6] National Research Council (US) Committee on A Framework for Developing a New Taxonomy of Disease. Toward Precision Medicine: Building a Knowledge Network for Biomedical Research and a New Taxonomy of Disease. Washington (DC): National Academies Press (2011). 22536618

[7] Turner MA Catapano M Hirschfeld S Giaquinto C. Global research in paediatrics. paediatric drug development: the impact of evolving regulations. Adv Drug Deliv Rev. (2014) 73:2–13. 10.1016/j.addr.2014.02.003 24556465

